# Hypersensitivity to fluoroquinolones: The expression of basophil activation markers depends on the clinical entity and the culprit fluoroquinolone

**DOI:** 10.1097/MD.0000000000003679

**Published:** 2016-06-10

**Authors:** Tahia D. Fernández, Adriana Ariza, Francisca Palomares, María I. Montañez, María Salas, Angela Martín-Serrano, Rubén Fernández, Arturo Ruiz, Miguel Blanca, Cristobalina Mayorga, María J. Torres

**Affiliations:** a Research Unit for Allergic Diseases, IBIMA-Regional University Hospital of Malaga-UMA, Málaga, Spain.; b Allergy Unit, IBIMA-Regional University Hospital of Malaga-UMA, Málaga, Spain.

**Keywords:** anaphylactic shock, anaphylaxis, basophils, fluoroquinolones, immediate hypersensitivity reactions

## Abstract

Although fluoroquinolones (FQs) are generally well-tolerated antibiotics, increasing numbers of hypersensitivity reactions have been reported. These can be evaluated in vitro by basophil activation tests (BATs); however, sensitivity is not optimal. Many factors could influence sensitivity such as basophil activation markers. The objective of this study was to evaluate the influence of 2 different activations markers, CD63 and CD203c, on the sensitivity of BAT to FQ. We studied 17 patients with immediate allergic reactions to FQ. BAT was performed with moxifloxacin and ciprofloxacin using CD193 (CCR3) for basophil selection and CD203c or CD63 as activation markers. Stimulation with ciprofloxacin induced a significantly higher expression of CD63 in ciprofloxacin-allergic patients compared to moxifloxacin-allergic patients (*P* = 0.002). In patients allergic to moxifloxacin with anaphylactic shock, we have observed an increase in the percentage of cells that upregulate CD203c, whereas patients with anaphylaxis preferentially upregulate CD63. The best sensitivity–specificity was obtained using a cutoff of 3 and the culprit FQ, using CD203c for moxifloxacin-allergic patients (sensitivity = 36.4%; specificity = 94.4%), and CD63 for ciprofloxacin-allergic patients (sensitivity = 83.3%; specificity = 88.9%). A negative correlation was found between the upregulation of CD63 and CD203c and the time interval between the reaction occurrence and the performance of the test (Spearman *r* = −0.446; *P* < 0.001 for CD63 and Spearman *r* = −0.386; *P* < 0.001 for CD203c). The performance of BAT for FQ allergy must be optimized for each drug, taking into account possible differences in the stimulation mechanism that leads to the upregulation of different activation markers.

## Abstract 2

Supplemental Digital Content is available in the text

## 1 Introduction

Fluoroquinolones (FQs) are generally considered well-tolerated antibiotics^[1]^ and have been used for over 30 years to treat a wide range of infections. Their consumption is increasing, particularly for certain derivatives like ciprofloxacin (CIP) and more recently moxifloxacin (MOX).^[2]^ This has led to an increase in allergic reactions and they are now the non-betalactam antibiotics most frequently involved in allergic drug reactions.^[3–6]^ Most of these reactions are thought to be IgE-mediated, being anaphylaxis and urticaria the most frequently reported,^[7–12]^ but since the introduction of MOX, more severe reactions, such as anaphylactic shock, have been reported.^[8,13]^ This FQ has been shown to be involved in >60% of severe reactions.^[11]^

Immediate allergic reactions to FQs are difficult to diagnose, and the value of skin testing is controversial. Although some consider the test useful,^[6,9,14,15]^ most studies show that FQs induce false-positive results,^[7,10,16,17]^ probably because of the capacity of some FQs to directly induce histamine release.^[10,14]^ Therefore, the only test available to diagnose these patients is the drug provocation test (DPT), which is not risk-free^[7,11,12]^ especially for severe reactions and older patients. There is a strong need for a biological test with adequate sensitivity.

To this end, the sepharose radioimmunoassay (Sepharose-RIA) has been assessed by some groups for evaluating immediate reactions to FQs; however, sensitivity ranges from 30% to 55%,^[7,11]^ results that can be explained by differences in the FQ involved and the severity of the reaction. More recently, studies have shown the utility of the basophil activation test (BAT) for evaluating FQ-allergic reactions^[6,10,11,18,19]^ with sensitivity ranging from 50% to 100%,^[11,18]^ which can be explained by different factors. One of these factors is the type of reaction that, as occurs with Sepharose-RIA, could influence the results, being in some studies mainly anaphylactic shock,^[18]^ whereas in others, the reactions were less severe such as anaphylaxis and urticaria.^[6,11]^ Sensitivity is also affected by both the FQs involved in the reaction and the FQs used for the test. It has been observed that basophil activation is higher with CIP than with MOX,^[11]^ which was in part explained by the FQs that originally sensitized the patients.^[11]^ However, it can also be explained by other intrinsic technical issues such as patient treatment, that is, systemic steroids and cyclosporin A.^[20,21]^ In addition, it can be influenced by the fast photodegradation of MOX, even in laboratory conditions, which influences the formation of drug–protein conjugates interfering with basophil activation.^[22]^ Finally, another factor that can influence the FQ BAT results could be the choice of activation markers, CD203c^[18]^ and CD63,^[10,11,22]^ as has been demonstrated for amoxicillin^[23]^ and neuromuscular blocking agents.^[24]^

Of the different basophil activation markers identified, CD63, a member of the transmembrane-4 superfamily anchored in the intracellular granules that are exposed in cellular surface after the degranulation process,^[25]^ is the most highly validated,^[25,26]^ although CD203c, constitutively expressed on the surface of resting basophils^[27]^ and upregulated after stimulation,^[28]^ has been also shown to be reliable.^[23,29]^ Both markers are related with degranulation and histamine release, but with 2 different mechanisms proposed, anaphylactic and piecemeal. The former is characterized by the fusion of the granules to the plasma membrane to expel their contents and leads to the exposition of CD63^[30]^; in piecemeal degranulation, small vesicles are formed from the histamine-containing granules and gradually shuttled to the plasma membrane. This is associated with the upregulation of CD203c.^[31]^ It is also possible that different drugs interact with basophils in different ways, activating 1 of these 2 pathways specifically. Another difference between these 2 markers is that CD203c is constitutively expressed in basophils and increases in expression after activation, whereas CD63 is expressed only after activation.^[32]^ Both markers have advantages and disadvantages: CD203c is specific to basophils; however, the increase in its expression is sometimes difficult to assess, owing to low changes mainly when evaluating drug hypersensitivity, or the induction of nonspecific increases when using interleukin-3 for priming basophils,^[33]^ as well as aspects of cells manipulation during the testing procedure.^[30]^

On the contrary, CD63 shows a bimodal expression, making it easier to measure; however, it is expressed not only in basophils but also in platelets.

In this study, we evaluated basophil response for 17 patients with confirmed immediate allergic reactions to FQs using 2 different activation markers CD63 and CD203c. We find that the upregulation of these markers depends on both the FQs used and the severity of the reaction.

## 2 Methods

### 2.1 Patients and controls

In this retrospective study, all patients referred to the Allergy Service of Regional University Hospital of Malaga, over a 3-year period (2013–2015) with an immediate allergic reaction after administration of a FQ derivative, were initially eligible for inclusion. Different clinical categories were established: anaphylaxis and anaphylactic shock, defined according to the criteria of Sampson,^[34]^ and urticaria when manifestations were limited to the skin and consisted of pruritic, erythematous cutaneous elevations that blanched with pressure at various sites on the body. In the allergological work-up, skin testing was not performed because of its low sensitivity and specificity.^[35]^ For ethical reasons, patients with anaphylaxis and anaphylactic shock were considered allergic from clinical history, once other possible causes were ruled out. In those with urticaria, a DPT was performed to confirm the diagnosis. The controls consisted of 18 cases with confirmed good tolerance to quinolones. None of the patients or controls received treatment with systemic corticoids or cyclosporine during the study.

The study was conducted according to the declaration of Helsinki and all patients and controls participating in the study gave their informed consent and protocols were approved by institutional ethical committees (Ethical Committee of Malaga).

### 2.2 DPT

Single-blind placebo-controlled DPT was carried out using CIP (Ciprofloxacino Normon, Madrid, Spain) or MOX (Actira, Bayer, Barcelona, Spain) under strict hospital surveillance, as described.^[11]^ The suspected quinolone was administered at 30-minute intervals in increasing doses until reaching the full therapeutic dose or symptoms of a drug reaction occurred. For CIP, the doses administered were 5, 50, 100, 150, and 200 mg (cumulative dose of 505 mg), followed by ambulatory therapeutical doses for 2 days and for MOX 5, 50, 100, 100, and 150 mg (cumulative dose of 405 mg), and followed by ambulatory therapeutical doses for 2 days.

### 2.3 Basophil activation by flow cytometry

BAT was a in-house-made test performed as previously described^[36]^ with different concentrations of quinolones: MOX at 0.2 and 0.1 mg/mL, and CIP at 2 and 0.2 mg/mL (all from Sigma Aldrich, Saint Louis, MO). The optimal concentrations for each drug were chosen based on dose–response curves and cytotoxicity.^[11]^ The samples were incubated while protected from light for 30 minutes at 37°C in a water bath to prevent FQ photodegradation.^[22]^ Cells were stained with monoclonal antibodies, anti-CD63-FITC, CD203c-PE, CCR3-APC (Caltag Laboratories, Burlingame, CA) and acquired in a FACSCalibur flow cytometer (Becton-Dickinson Bioscience, San Jose, CA) by acquiring at least 500 to 1000 basophils per sample, selected as CCR3^+^ cells (Fig S1). Results were analyzed at blind by 2 independent well-trained researchers with FlowJo software (FlowJo LLC, Ashland, OR) and activation expressed as percentage of CD63 or percentage of CD203c and as stimulation index (SI). SI was calculated as the ratio between the percentage of activated basophils (CD63^+^ or CD203c^+^ cells) in samples stimulated with the different haptens and the unstimulated sample. The percentage of spontaneously activated basophils (unstimulated sample) was required to be ≥5% to calculate the SI, as previously described.^[36]^

### 2.4 Statistical analysis

Comparisons of quantitative variables without a normal distribution were done by the Mann–Whitney and Kruskall–Wallis tests. Comparisons of qualitative variables were done by means of the *χ*^2^ test. Correlation was measured using Spearman rank correlation coefficient. All reported *P* values represent 2-tailed tests, with values <0.05 considered statistically significant. The R Project software 3.1.2 was used for the analysis.

## 3 Results

The study included 17 patients with confirmed immediate allergic reactions to FQ (Table 1). Thirteen were women (76.5%) and the median age was 65 (interquartile range [IR]: 48–80) years. The median time interval between the reaction and the study was 11.12 (IR: 1–84) months. The drugs involved were MOX for 11 (64.7%) and CIP for 6 (35.3%) patients. The clinical entities observed were anaphylactic shock in 5 (29.4%), anaphylaxis in 7 (41.2%), and urticaria in 5 cases (29.4%). Significant differences in clinical manifestation were found in different groups depending on the culprit FQ (*P* = 0.006) as in all patients with anaphylactic shocks the culprit FQ was MOX (45.5%). In those cases with urticaria, the diagnosis was confirmed by a DPT. A control group of 18 sex- and age-matched tolerant subjects was also included.

**Table 1:**
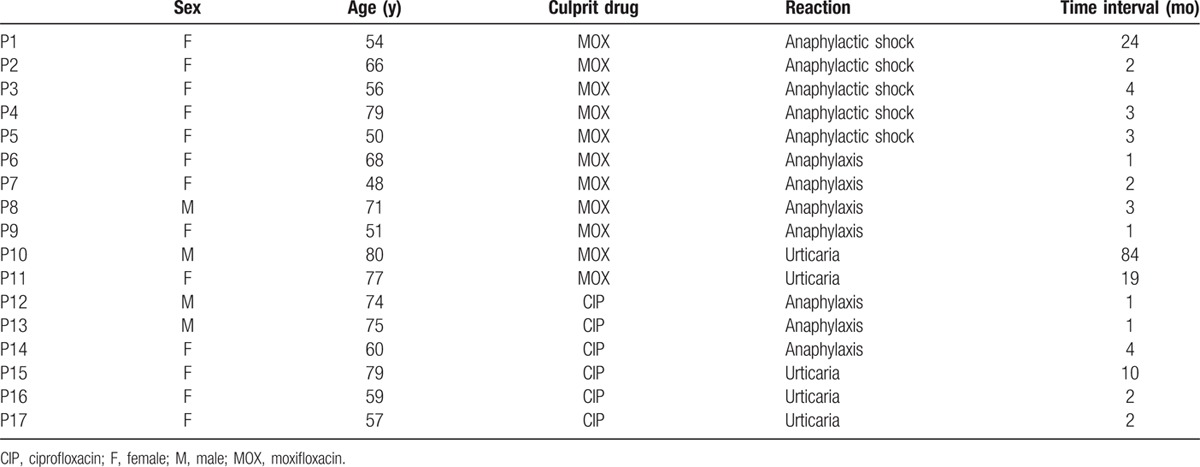
Clinical characteristics of patients included in the study.

### 3.1 CD63 and CD203c upregulation

**Figure 1:**
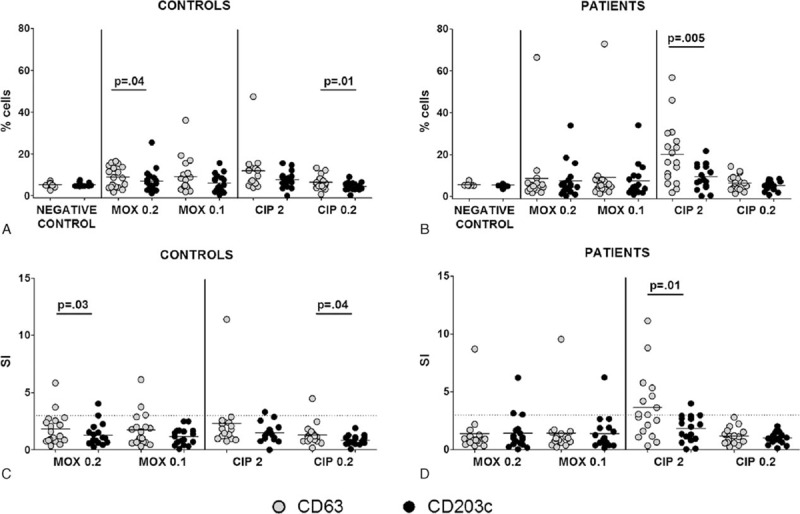
Basophil activation test (BAT) results in fluoroquinolone (FQ)-allergic patients and controls. Comparison of expression levels for CD63 and CD203c as (A) percentage of activated cells in controls; (B) stimulation index (SI) in controls; (C) percentage of activated cells in FQ-allergic patients; (D) SI in FQ-allergic patients, represented as individual data points. Lines represent the mean of all data. Wilcoxon matched-pair tests were performed.

Higher expression of CD63 was observed for all FQs and concentrations tested, although differences were only significant at 0.2 mg/mL for CIP and MOX when data were analyzed in terms of percentage (*P* = 0.04 for MOX; *P* = 0.01 for CIP) (Fig. 1A) and SI (*P* = 0.03 for MOX; *P* = 0.04 for CIP) (Fig. 1C). In allergic patients, CIP could upregulate both CD63 and CD203c, although the percentage of cells expressing CD63^hi^ was significantly higher compared to CD203c^hi^ (*P* = 0.005). These differences were not detected with MOX (Fig. 1B). Equivalent results were observed for SI (*P* = 0.01) (Fig. 1D).

**Figure 2:**
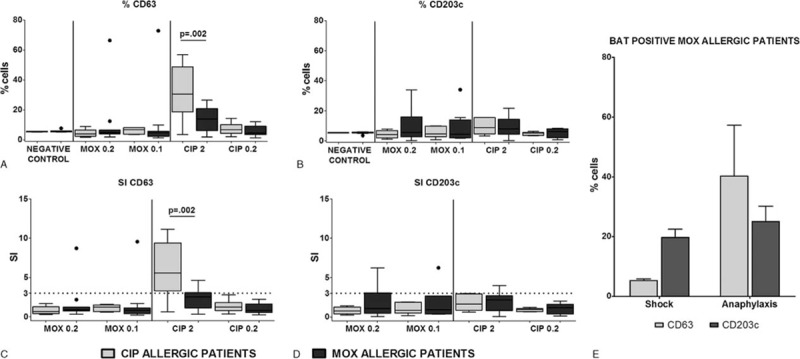
Comparisons of BAT results in CIP and MOX allergic patients as (A) percentage of cells expressing CD63 or upregulating CD203c and (B) stimulation index (SI) calculated with %CD63 and %CD203c. Box plots represent the median and IQR. Statistical Mann-Whitney U tests were performed. (C) Differences in activation marker up-regulation in BAT positive MOX allergic patients. Bars represent the mean and SEM of the percentage of cells expressing CD63 or CD203c in MOX allergic patients with positive BAT, discriminating between the types of reaction: Anaphylactic Shock or Anaphylaxis.

Classifying the patients according to the culprit FQ, we observed a significantly higher basophil expression of CD63 after CIP stimulation in patients allergic to this FQ (*P* = 0.002) compared with MOX-allergic patients (Fig. 2A), with similar results found using SI (*P* = 0.002) (Fig. 2B). Regarding CD203c, the highest values were obtained for MOX-allergic patients using the same FQ for the test, although the differences were not significant (Fig. 2A and B).

In terms of the relation between the upregulated marker and the clinical entity, we analyzed MOX-allergic patients, the only group that included patients suffering from anaphylactic shock, anaphylaxis, and urticaria. We observed an increase in the percentage of cells that upregulate CD203c in the patients with anaphylactic shock and in the percentage of cells that upregulate CD63 in patients with anaphylaxis (Table 2), although these differences were not significant. However, when the same analysis was done including only positive patients, we observed a higher increase in CD203c in the anaphylactic shock patients compared with CD63, whereas in patients suffering from anaphylaxis, we observed an increase in CD63 cells (Fig. 2C). No positive BAT was found in urticaria patients. Moreover, we compared the expression of activation markers, CD63 and CD203c, in the 2 most frequent clinical entities, anaphylaxis and urticaria, obtained after incubation with their respective culprit FQ. Data showed a higher expression of CD63 independently of the clinical entities and the FQ involved in the reaction (Fig. S2).

**Table 2:**
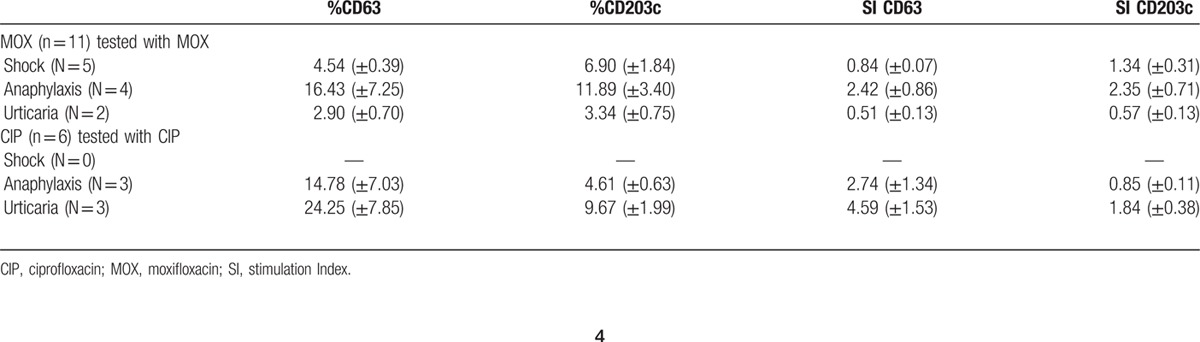
BAT results in moxifloxacin and ciprofloxacin allergic patients. Data represent means ± SEM.

### 3.2 Sensitivity and specificity

We assessed 2 different SI cutoff points, 2 and 3, with results showing a better sensitivity–specificity with a cutoff of 3 and using the culprit FQ. Using CD203c as activation marker for MOX-allergic patients gave sensitivity = 36.4%; specificity = 94.4%; using CD63 for CIP-allergic patients gave sensitivity = 83.3%; specificity = 88.9% (Table 3).

**Table 3:**
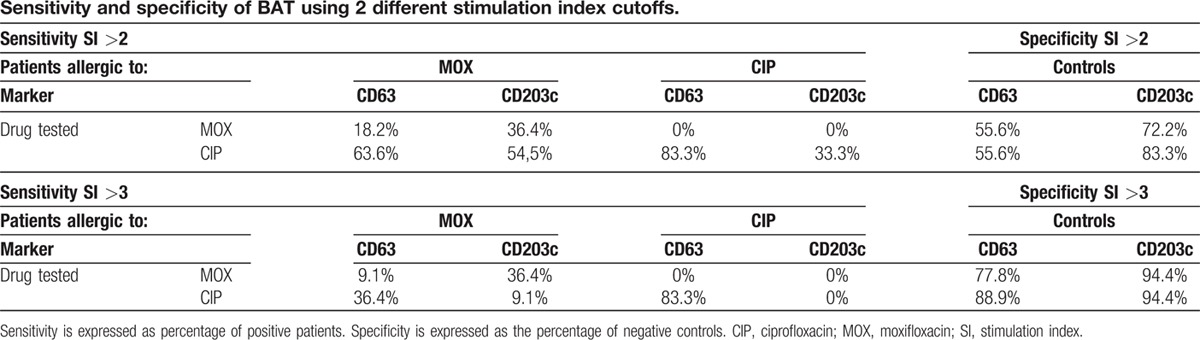
Sensitivity and specificity of BAT using 2 different stimulation index cutoffs.

### 3.3 Correlation between CD63 and CD203c activation markers

**Figure 3:**
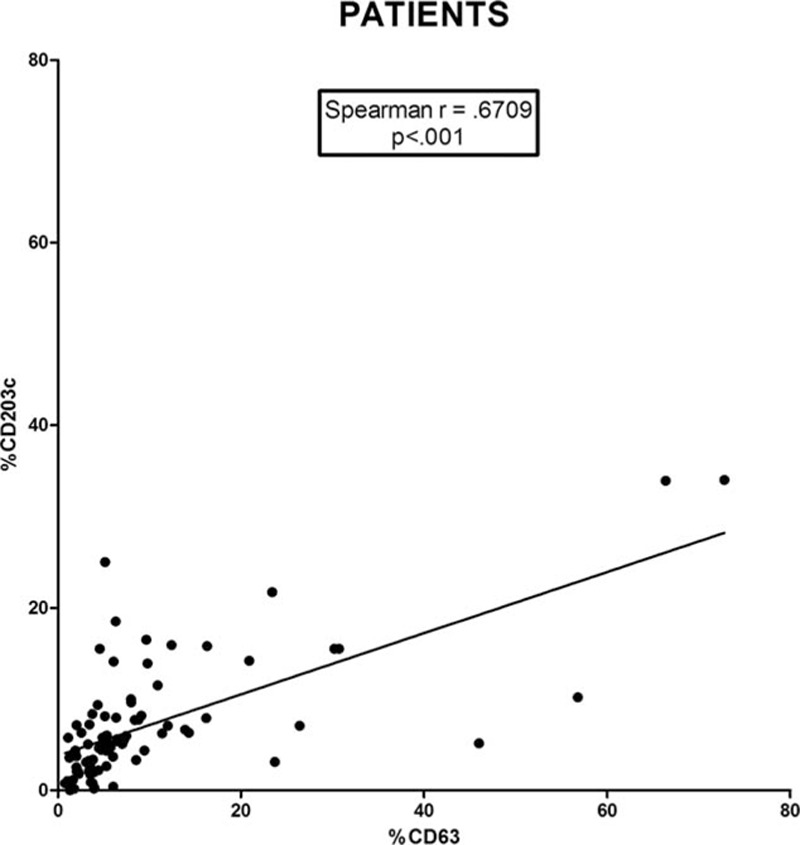
Correlation between CD63 and CD203c expression in FQ-allergic patients. Each point represents an individual patient for a given drug concentration.

Expression of both markers, CD63 and CD203c, showed a positive correlation (Spearman *r* = 0.671; *P* < 0.001) in the whole group of FQ-allergic patients, indicating that both are valid markers in BAT (Fig. 3). Significant correlations between the 2 markers were also obtained for MOX-allergic patients (Spearman *r* = 0.725; *P* < 0.001) (Fig. S3A) for anaphylactic shock (Spearman *r* = 0.661; *P* = 0.007) (Fig. S3B) and anaphylaxis (Spearman *r* = 0.923; *P* < 0.001) (Fig. S3C). The slope was more pronounced in the anaphylactic shock patients showing the relevance of CD203c in this diagnosis.

We have also analyzed the effect of the time interval between the reaction occurrence and the performance of the test in the upregulation of basophil activation markers, finding a negative correlation for both markers (Spearman *r* = −0.446; *P* < 0.001 for CD63 and Spearman *r* = −0.386; *P* < 0.001 for CD203c) (Fig. S4A and S4B). This seems to be more important for MOX-allergic patients, and their best marker CD203c, with a higher negative correlation with the time interval (Spearman *r* = −0.646; *P* < 0.001) (Fig. S4C).

## 4 Discussion

In the last decade, increased consumption of FQs has led to more allergic reactions to these drugs and increased severity.^[3,8,11]^ Diagnosis is difficult owing to the low value of skin testing,^[6,7,9,10,14–17]^ as some FQs have the capacity to induce direct histamine release.^[17]^ In fact, it has been described that a mast cell receptor, MRGPRX2, can be activated nonspecifically by FQs, inducing degranulation and release of histamine, β-hexosaminidase, tumor necrosis factor, and PGD_2_ among others, being probably one of the reason for false-positive results in skin tests.^[37]^ BAT with FQ^[6,10,11,18]^ has been used for diagnosis with sensitivity ranging from 50%^[19]^ to 100%^[18]^ and specificity from 80%^[11]^ to 100%.^[6,18]^ These differences may be because of the number of patients,^[10,18]^ culprit FQ, the clinical manifestations, with only 2 studies including patients with anaphylactic shock,^[11,18]^ and the activation markers used. In this work, we have analyzed 2 different basophil activation markers, CD63 and CD203c, to diagnose immediate reactions to MOX and CIP in a well-characterized population with a high proportion of severe reactions, including anaphylaxis and anaphylactic shock (70.6% of cases). As reported in previous studies,^[8,11,12]^ we have detected that anaphylactic shock only occurred in MOX-allergic patients (45.5% of these reactions). Interestingly, we have found that the use of each FQ, MOX, or CIP in the BAT mainly induces the upregulation of a specific activation marker, CD63 or CD203c, which could be related to the severity of the reaction; however, further analysis with larger number of patients should be carried out to confirm this observation.

Although mast cells have been considered the primary effector cells in IgE-mediated reactions, basophils also play an important role in the development of an early immune response^[38,39]^ and constitute an important target for the in vitro evaluation of these reactions. CD63, the most widely used activation marker,^[25]^ is exposed at the cellular surface after the degranulation processes,^[25]^ whereas CD203c is constitutively expressed on the surface of resting basophils^[27]^ and upregulated after stimulation.^[28]^ Although both markers were correlated in expression when considering all patients, our results showed differences related to the FQs used in the test. We found that CIP induced a greater upregulation of CD63, whereas MOX preferentially upregulated CD203c. A similar pattern can be found for other drugs: Abuaf et al^[23]^ (2008) found that CD203c seems to have more sensitivity for the detection of amoxicillin-activated basophils, but CD63 had more sensitivity in patients with anaphylaxis to muscle relaxants.^[24]^ Differences have also been described for allergens: CD203c is a better marker for latex allergy^[40]^; CD63 is more specific but CD203c more sensitive for bee or wasp allergy^[32]^; and both can be reliable markers for cat allergy diagnosis.^[29]^ These studies suggest that different basophil activation pathways exist depending on the culprit.

Other explanations for these differences could be based on the existence of different activation mechanisms related to the clinical entity. Analyzing MOX-allergic patients, the only group containing patients suffering from all 3 clinical entities, we have observed differences in the upregulated marker: in patients who suffered anaphylactic shock, an upregulation of CD203c was found, whereas patients with anaphylaxis showed a CD63 upregulation. These markers are differentially located in the cell, CD203c molecules in vesicles near the membrane, whereas CD63 is stored in granules.^[32]^ These molecules might be related to the 2 potential degranulation pathways, piecemeal and anaphylactic. In piecemeal degranulation, small vesicles from the histamine-containing granules are formed and rapidly shuttled to the plasma membrane.^[30,31]^ This process could lead to the upregulation of CD203c, present in other small vesicles distinct from histamine-containing granules,^[33]^ and may be linked to stimulation by certain drugs and the development of severe reactions like anaphylactic shock.^[18]^ In the second mechanism, anaphylactic degranulation, the main granules are fused to the plasma membrane, releasing the entire contents to the extracellular space. CD63, present in the membrane of these granules, is thus exposed on the surface of basophils.^[33]^ This process is slower than piecemeal degranulation and could be related to the development of anaphylaxis or urticaria.^[8]^

The time interval between reaction occurrence and the BAT performance should also be considered. We have found negative correlations between the 2 markers, CD63 and CD203c, and the time interval. This agrees with results for other drugs, wherein a rapid negativization of the test has been found several months after the reaction occurred, owing to the loss of drug-specific IgE in non-reexposed patients,^[41,42]^ and highlights the importance of performing BAT soon after the reaction.

Finally, we found better results for sensitivity and specificity using an SI cutoff of 3 and using the culprit drug for the test. However, the activation marker must be different for each drug: CD203c for MOX- and CD63 for CIP-allergic patients. Moreover, it is important to take into account the type of reaction: it is important to include CD203c in addition to CD63 when evaluating anaphylactic shock, whereas CD63 seems to be more related to anaphylactic reactions, as has been previously reported.^[11]^

In conclusion, the performance of BAT for drug allergy must be optimized for each drug, taking into account the possible differences in the stimulation mechanism that leads to the upregulation of different activation markers, influencing BAT results.

## Acknowledgements

The authors thank James R Perkins for help with English language, Ana Molina for help with the laboratory work and IBIMA major statistics service for help with statistical analysis.

## Supplementary Material

Supplemental Digital Content
