# Supplementary material for: Hypersensitivity to fluoroquinolones: The expression of basophil activation markers depends on the clinical entity and the culprit fluoroquinolone
Source: Medicine (Baltimore). 2016 Jun 10;95(23):e3679. doi: 10.1097/MD.0000000000003679 (PMC4907647; doi:10.1097/MD.0000000000003679)
Supplement: Supplemental Digital Content [file medi-95-e3679-s001.docx]

**
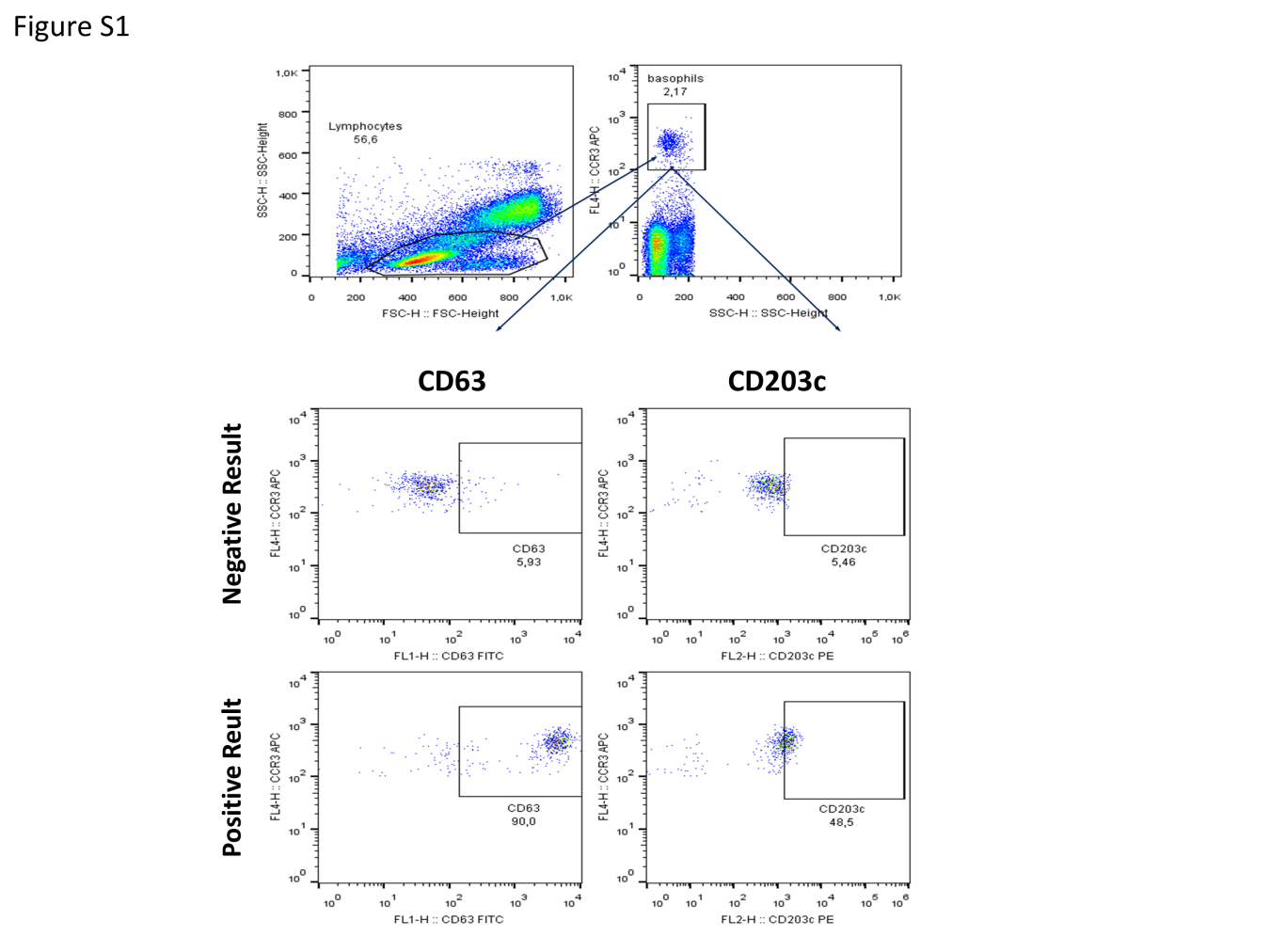
**

**Figure S1. Flow cytometry gating strategies for the different subpopulations analyzed.** Basophils were selected as CCR3^high^ cells from the lymphocyte population. The percentage of spontaneously expression of CD63 or CD203c was required to be equal to or greater than 5% to gate the positive population.

**
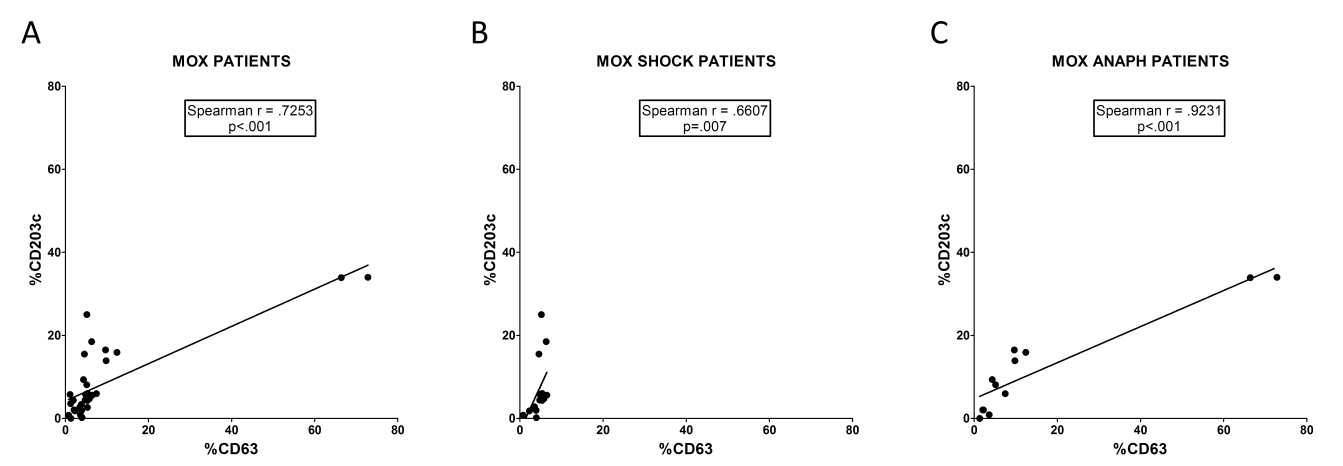
**

**Figure S2. Correlation between both activation markers, CD63 and CD203c:** A) in MOX allergic patients; B) in MOX allergic patients that suffered anaphylactic shock; C) in MOX allergic patients that suffered anaphylaxis. Each point represents an individual patient for a given drug concentration.


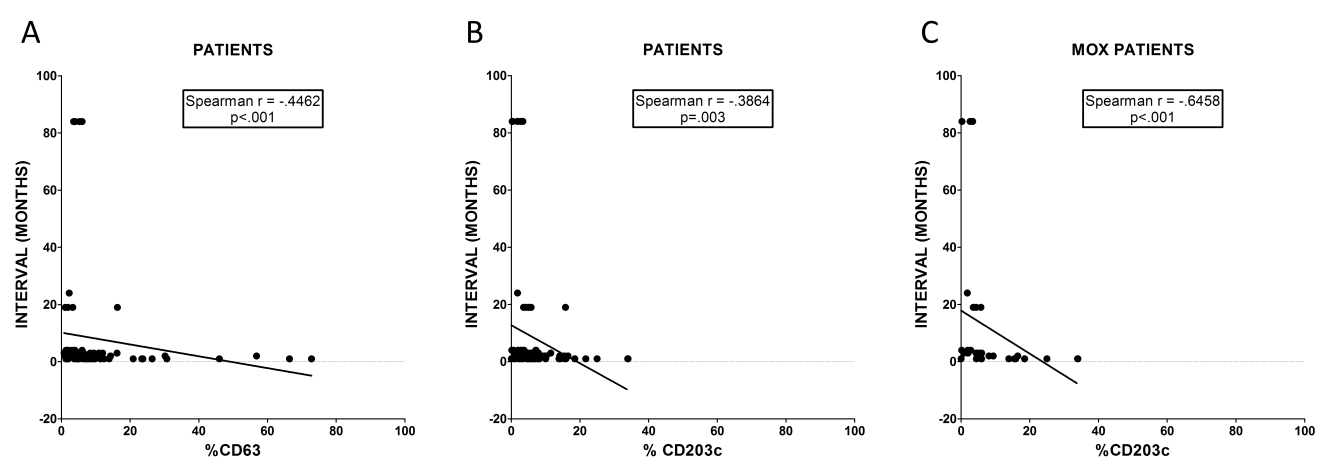


**Figure S3: Correlation between activation markers and the time interval:** A) with CD63 in the whole group of patients; B) with CD203c in the whole group of patients; C) with CD203c in MOX allergic patients. Each point represents an individual patient for a given drug concentration.
